# Atypical erythema multiforme

**DOI:** 10.11604/pamj.2015.20.436.5882

**Published:** 2015-04-30

**Authors:** Fatima Zahra Elfatoiki, Soumia Chiheb

**Affiliations:** 1 Department of Dermatology, Ibn Rochd UHC of Casablanca, Morocco

**Keywords:** Genital erythematous, target-like lesions, apoptotic individual keratinocytes

## Image in medicine

4-year-old child presented oral and genital eruptions for 2 days, he had history of herpetic stomatitis 3 days before. Examination revealed oral painful erosive lesions with genital erythematous purpuric patch mimiking atypical target-like lesions. No others cutaneous lesions were observed. Skin biopsy showed apoptotic individual keratinocytes, degeneration of basal keratinocytes and intercellular oedema, it concluded to diagnosis of erythema multiforme. The patient was treated by Aciclovir 5mg/kg/day for 1 week with oral antihistamines, analgesics and local skin care. A complete resolution was noted after 2 weeks.

**Figure 1:**
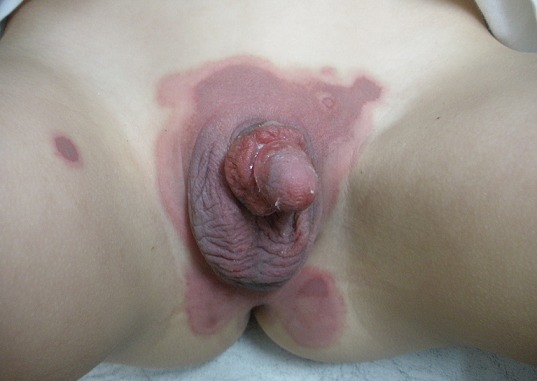
Génital atypical target-like lesions

